# American Dental Association

**Published:** 1885-09

**Authors:** 


### EDITORIAL.

### American Dental Association.

### TWENTY-FIFTH ANNUAL SESSION.

Crossing the Mississippi river for the first time since its organization in 1859, the twenty-fifth annual meeting of the American Dental Association, held at Minneapolis, August 4th to 7th, inclusive, was one of the best meetings ever held, in point of prominent members present, of new members admitted, of non-members in attendance, in scientific and practical value of papers read, and in interest and animation of discussion.

Two hundred and fifty-eight members paid their dues, with the unprecedented number of 103 new members enrolled.

The offices were all present, punctually, at their posts.

The dental journals and the local press were represented by an unusual number of reporters. Quite a number of ladies were in attendance. The number of young dentists was quite as remarkable, as also the number of men of portly presence.

Several rooms adjacent to the Hall, were occupied by a fine display of dental instruments, appliances and materials.

Over 200,000 teeth were on exhibition, about 100,000 being from the S. S. White Manufacturing Company.

The report of the Treasurer, at the opening of the meeting, showed a balance on hand of nearly $2,000, which was largely increased before the adjournment. In view of this very satisfactory financial state of affairs, it was voted to appropriate the sum of $200, each to sections v, vi and vii, for the prosecution of original scientific investigation and research.

Equal or smaller amounts might with propriety and promise of good results have been appropriated to the other sections.

The secretary announced the death since the last meeting of Dr. J. G. Ambler, of New York, and Dr. Isaiah Forbes, of St. Louis. Suitable memorial resolutions were adopted.

A resolution was also passed in memory of General Grant.

The first paper read was presented by Sec. vii, Physiology and Etiology. Subject:—The Earthy Phosphates, by Dr. W. C. Barrett. This was an elaborate argument on physiological grounds, against the administration of the inorganic phosphates, maintaining that all inorganic substances must be elaborated and organized by the vegetable kingdom before they could be assimilated into animal tissues.

On this subject we may have something to say at another time.

Section iii. Dental Literature and Nomenclature, reported two papers, one from Dr. Atkinson and one from Dr. Kulp.

Dr. Kulp, of Iowa, read a very practical paper, systematically classifying the teeth and their surfaces, so that each term conveys a definite idea, clearly locating every possible cavity, in terms brief and definite.

Section iv. Operative Dentistry reported one paper. The painless operation by Dr. J. A. Robinson, of Jackson, Mich., and several subjects for discussion, viz., Bridge Work, the Herbert method, the Perry Separators and Matrices, Half-bicuspid Crowns, and an appliance to regulate the exhalations of patients.

Dr. E. Q. Brown gave some description of bridge work, illustrating by specimens. Dr. Kulp also spoke of the advantages of bridge work when properly done.

Quite a discussion was had upon the use of the matrix in filling teeth. It was strongly recommended by some for all proximate filling, while others recommended discrimination and caution in its use.

Dr. Southwell, of Milwaukee, exhibited a very ingenious device to protect the operator from the patient's breath.

The entire afternoon of Wednesday was devoted to clinics, under the arrangements provided by the committee: Drs. A. T. Smith, Gardner, and Geo. L. Field.

The clinic was an interesting one, in which Dr. Timmer, of Hoboken, and Rhein, of New York, demonstrated the Herbest method of filling. Drs. Brown, Noyes, Moore, and St. John filled some difficult cavities in a superior manner.

Dr. Harlan treated cases of Pyorrhea Alveolaris. Indeed there was so much done that we must refer to the full report.

The report of Section v, Anatomy, Histology, and Microscopy, was read by Prof. Frank Abbott, in which was presented "Studies of the Pathology of Enamel of Human Teeth, with Special Reference to the Etiology of Caries," and by Prof. L. C. Ingersoll, a paper on "The Dental Alveolar Membrane, Unity or Duality, which ?"

Both of these papers were exhaustive on their respective subjects. Prof. Ingersoll made, seemingly, quite a conclusive argument in favor of the proposition that the dental periosteum is "dual."

The papers were read from Sec. vi. And also from Sec. i.

We have not space here to make further reference to the work done at this meeting; but will refer the reader to the October number of the REGISTER which will contain a full report of the proceedings, including a synopsis of the papers read and the discussions upon them. This will repay a careful reading.

